# Crosstalk between Nitrite, Myoglobin and Reactive Oxygen Species to Regulate Vasodilation under Hypoxia

**DOI:** 10.1371/journal.pone.0105951

**Published:** 2014-08-22

**Authors:** Matthias Totzeck, Ulrike B. Hendgen-Cotta, Malte Kelm, Tienush Rassaf

**Affiliations:** Department of Medicine, Division of Cardiology, Pulmonary Diseases and Vascular Medicine, Medical Faculty, University Hospital Düsseldorf, Düsseldorf, Germany; Albany Medical College, United States of America

## Abstract

The systemic response to decreasing oxygen levels is hypoxic vasodilation. While this mechanism has been known for more than a century, the underlying cellular events have remained incompletely understood. Nitrite signaling is critically involved in vessel relaxation under hypoxia. This can be attributed to the presence of myoglobin in the vessel wall together with other potential nitrite reductases, which generate nitric oxide, one of the most potent vasodilatory signaling molecules. Questions remain relating to the precise concentration of nitrite and the exact dose-response relations between nitrite and myoglobin under hypoxia. It is furthermore unclear whether regulatory mechanisms exist which balance this interaction. Nitrite tissue levels were similar across all species investigated. We then investigated the exact fractional myoglobin desaturation in an *ex vivo* approach when gassing with 1% oxygen. Within a short time frame myoglobin desaturated to 58±12%. Given that myoglobin significantly contributes to nitrite reduction under hypoxia, dose-response experiments using physiological to pharmacological nitrite concentrations were conducted. Along all concentrations, abrogation of myoglobin in mice impaired vasodilation. As reactive oxygen species may counteract the vasodilatory response, we used superoxide dismutase and its mimic tempol as well as catalase and ebselen to reduce the levels of reactive oxygen species during hypoxic vasodilation. Incubation of tempol in conjunction with catalase alone and catalase/ebselen increased the vasodilatory response to nitrite. Our study shows that modest hypoxia leads to a significant nitrite-dependent vessel relaxation. This requires the presence of vascular myoglobin for both physiological and pharmacological nitrite levels. Reactive oxygen species, in turn, modulate this vasodilation response.

## Introduction

Hypoxic vasodilation is one of the key adaptive responses to maintain an equilibrium between oxygen (O_2_) supply and demand for e.g. muscle tissue at work [1]. Although the observation that vessels dilate when subjected to decreasing O_2_ tensions was made more than 150 years ago, the underlying signal transduction mechanisms have remained under intensive debate [2], [3]. This pertains both to the O_2_ sensor as well as to the coupled vasodilatory effector signaling. It is generally believed that upon reaching a critical O_2_ saturation, a sensor mechanism transmits this event to a cellular signaling cascade, which finally decreases the levels of intracellular [Ca^2+^] with subsequent relaxation of the vascular smooth muscle machinery [4]. Many different mechanisms have been proposed to contribute to these processes including adenosine, pH changes, prostacyclin and potassium [5]–[8]. The exact nature of the vasodilatory mechanism, however, remained unresolved.

Vasodilation under normoxia is mediated by the release of nitric oxide (NO^•^) from the endothelial NO synthase (eNOS) through e.g. enhanced shear stress, and relies on the availability of an intact endothelium [9]. This activates the canonical NO^•^/soluble guanylate cyclase (sGC)/cyclic guanosine monophosphate (cGMP) pathway finally decreasing intracellular [Ca^2+^] [10]–[12]. eNOS operates under normoxic conditions and its activity decreases with low O_2_ tensions. eNOS was therefore regarded to be an unlikely source for vasodilatory signaling under hypoxia.

We and others have recently demonstrated that nitrite, once regarded to be an oxidative end-product of NO^•^ breakdown, is a source for bioactive NO^•^ under hypoxia [13]–[16]. Nitrite is reduced to NO^•^ along physiological and pathophysiological O_2_ and pH gradients in the circulation and a wide variety of tissues. A number of mechanisms have been forwarded for this bioactivating process including xanthine oxidoreductase, hemoglobin (Hb), myoglobin (Mb), neuroglobin, cytoglobin and even eNOS [9], [13], [17]–[21]. The relative contribution of each reductase appears to depend on the corresponding tissue, the exact O_2,_ pH and nitrite levels [22]. Remarkably, the concentration of nitrite in tissues differs significantly from those described for the circulatory compartment with levels being consistently and up to >20 times higher in solid tissues, e.g. in the vasculature [23], [24]. The mechanisms of nitrite uptake into the cellular compartment and the maintenance of a nitrite equilibrium and particular a high tissue stability of the described levels remain a matter of current investigations [25]. It was recently demonstrated in hypoxia-tolerant fish that sustained exposure to anoxia dramatically increases the intracellular levels of nitrite while reducing those in the systemic circulation [26]. On the contrary, experiments in rodents under hypoxia have suggested a relevant reduction in vascular nitrite levels implicating a potential consumption [27].

The fact that higher nitrite levels dilate isolated vessel preparations has been detected in the early 1950s by Furchgott et al [28]. In 2003, Cosby and coworkers demonstrated that near physiological nitrite levels are capable of vasodilation given the presence of a partially deoxygenated nitrite-reductase – Hb [13]. Apart from its vasodilatory properties, very low nanomolar concentrations of nitrite were shown to have beneficial effects for cytoprotection under hypoxia/ischemia. In the heart, deoxygenated Mb, another member of the heme globin family, reduces nitrite to cardioprotective NO^•^, which then regulates mitochondrial function and protects the myocardium from lethal ischemia/reperfusion injury [9], [14], [29]. We and others have recently shown that Mb is also expressed in smooth muscle layers of mouse aorta [4]. We furthermore demonstrated that under hypoxia, vascular Mb reduces nitrite to NO^•^, which then activates the vasodilatory signaling machinery in smooth muscle cells, leading to hypoxic vasodilation and reduction of blood pressures independent of changes in cardiac functions [4]. Remarkably, this new mechanism for hypoxic vasodilation localizes the sensor (deoxygenated Mb) and effector signaling within the same smooth muscle cell. While we have previously demonstrated that this mechanism is effective under physiological baseline conditions and under higher pharmacological nitrite levels [4], the exact dose-response relationship between Mb and nitrite remains to be determined.

Recent studies have closely linked cytoprotection from nitrite to an interaction with reactive oxygen species (ROS) [29]–[31]. In the context of hypoxic vasodilation, ROS have also been characterized sensors for an increasing imbalance between the supply and demand for O_2_ and energy sources [32], [33]. ROS may also have a counteracting, modulating effect on isolated arteries, which was demonstrated by reduction of superoxide radical anion (O_2_^•−^) and hydrogen peroxide (H_2_O_2_) in isolated penile arteries [34]. Possible regulatory effects of ROS on nitrite induced hypoxic vasodilation have not been described so far.

We here sought to examine the (i) dose-response relationship between nitrite and Mb to dilate isolated vessels along physiological and pharmacological levels and whether (ii) ROS modulate this process.

## Materials and Methods

### Animals

NMRI (Naval Medical Research Institute, *Mb^+/+^* and *Mb^−/−^* [35]) and *eNOS^−/−^* [36] mice were obtained from the Düsseldorf animal house. All mice were male and the groups did not differ in age (12±3 weeks) or weight (32±6 g). Animals were held on standard chow and tap water *ad libitum* and on a 12/12 hours light/dark cycle. All experiments were approved by the responsible ethics committee according to the ‘European Convention for the Protection of Vertebrate Animals used for Experimental and other Scientific Purposes’ (Council of Europe Treaty Series No. 123).

### Chemicals

All chemicals were bought from Sigma (Seelze, Germany) except for phosphate-buffered saline (PBS, Serag-Wiessner, Naila, Germany), heparin (ratiopharm, Ulm, Germany), ketamine (Pfizer, Berlin, Germany), xylazine (aniMedica, Senden, Germany), isoflurane (DeltaSelect, Pfullingen, Germany), and acetylcholine (Fluka).

### Preparation of Mb protein solution and assessment of Mb desaturation

We used phosphate buffered saline (PBS, Serag Wiessner, Germany) for the preparation of all described Mb protein solutions. Horse Mb was incubated with excess sodium dithionite [14] and then gassed with 21% O_2_/79% nitrogen (Linde, Germany) at atmospheric pressure to receive oxygenated Mb (oxyMb). Photometric wavelength scans were performed to evaluate the relative presence of oxyMb. The absolute Mb levels were calculated using the extinction coefficients for oxyMb (ε_418nm_ = 128 mM^−1^ cm^−1^, ε_542nm_ = 13.9 mM^−1^ cm^−1^ and ε_580nm_ = 14.4 mM^−1^ cm^−1^) and for ferric Mb (ε_409nm_ = 153 mM^−1^ cm^−1^). Only pure oxyMb solutions were used for experiments. A 1% O_2_/99% nitrogen gas mixture was used to deoxygenate the Mb solution followed by spectrophotometrical assessment the generated deoxyMb using its destinct coefficient (ε_560nm_ = 13.8 mM^−1^ cm^−1^).

### Determination of vascular nitrite levels

Anesthesia was achieved by intraperitoneal injection of ketamine (45 mg/kg) and xylazine (Rompun, 10 mg/kg). After neck dissection and thoracotomy, aortic tissue was excised, freed from adipose tissue and immediately snap frozen. Vascular nitrite levels were assessed as previously described using HPLC-based technique (ENO-20) [4], [23], [24], [37]–[40].

### Aortic ring bioassay

2–3 rings of a single thoracic mouse aorta were suspended in an organ bath containing 10 ml Krebs-Henseleit buffer and connected to force transducers (Hugo Sachs). The bath was purged with 5% CO_2_/95% O_2_ and equilibration was allowed for 60 min. Resting tension was set to 1 g. Following addition of KCl (40 mM, final), viability was checked with 10 µM phenylephrine (final) followed by 10 µM acetylcholine (final). After a second preconstriction with phenylephrine, nitrite (0.1–1,000 µM) was added cumulatively and the relaxation was calculated as % of the maximum constriction. For hypoxia, aortic rings were equilibrated to 1% O_2_ for 20 min [41] and then challenged with cumulating nitrite doses. The effects on vasodilation/vasorelaxation were calculated as relative changes with 100% being maximum preconstriction and 0% representing complete vasodilation (resting tension).

For experiments concerning the role of ROS, aortic rings were incubated with 1 µM SOD, 200 U/ml catalase, 30 µM 4-Hydroxy-TEMPO (SOD mimetic, tempol) and/or 5 µM ebselen 10 min before the onset of hypoxia. After pre-constriction, vasodilation to 300 nM (which closely correlates to mouse plasma conditions) with and without ROS decomposition enzymes was measured. Tempol was used because of its superior tissue penetration as compared to SOD, which hardly penetrates cell membranes [42].

### Statistical analysis

Values represent means±s.d. or means±s.e.m. as indictated, with n independent experiments. Data were analyzed by Student's t-test and ANOVA (multiple groups) with Holm-Sidak correction for multiple experiments using Prism 6.0 (GraphPad). A value of P<0.05 was considered to be statistically significant. EC_50_s were log_10_ transformed before statistical comparison to achieve normal distribution.

## Results

### Mb fractional O_2_ desaturation

Nitrite, as derived from NO^•^ oxidation or from nutritional sources, represents a source of bioactive NO^•^ along physiological and pathological O_2_ gradients. A large body of evidence supports the notion of heme globin-related hypoxic nitrite signaling. Mb, as present in striated, cardiac and smooth muscles, is among the most potent nitrite reductases [43]. The half-saturation of Mb is much lower as compared to Hb suggesting a significant contribution at very low O_2_ tensions only with nearly desaturated Mb. Prior to assessing the dose-dependent effects of Mb-related nitrite reduction on vessel dilation under hypoxia, we set out to test whether exposure to a generally-acknowledged hypoxia level leads to a significant Mb desaturation.

**Figure 1:**
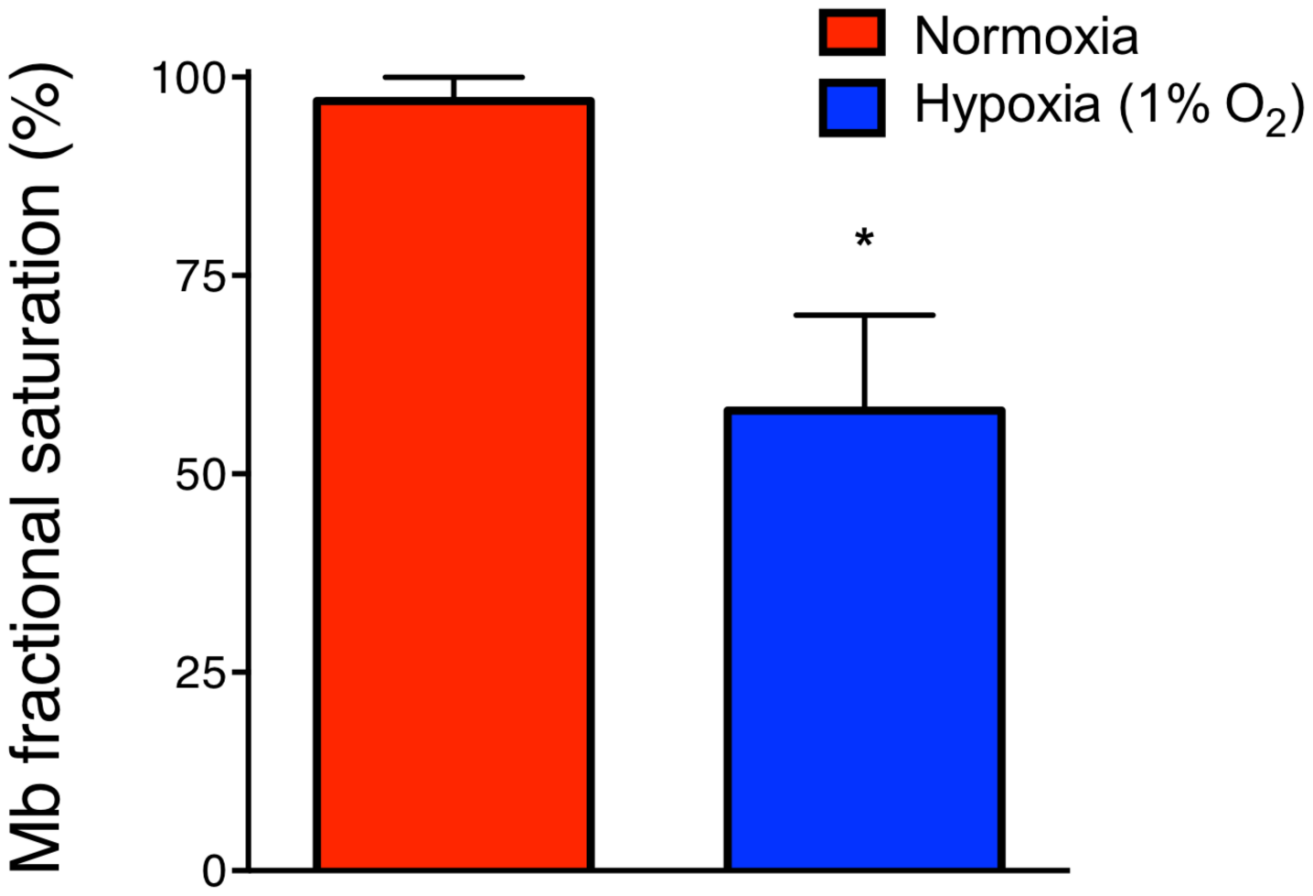
Hypoxia-induced myoglobin desaturation. Gassing with a 1% oxygen gas mixture leads to shift of the UV vis spectrum from oxygenated myoglobin (oxyMb) to deoxygenated myoglobin (deoxyMb). Figure shows final levels of saturation with a significant reduction under hypoxic gassing (means±s.d).

A 1% O_2_ gas mixture was used to simulate hypoxia in our experimental *ex vivo* approach [41], [44], [45]. UV vis spectroscopy was applied to explore the corresponding saturation of Mb resulting from the deoxygenation procedure. The fractional saturation hereafter was determined to be 58±12% (*n* = 3, *P*<0.05, **Figure 1**). We suggest that these measurements are generally important to underline a relevant abundance of Mb as well as heme globins in general for a significant contribution to nitrite reduction.

### Tissue nitrite levels across different mouse species

**Figure 2:**
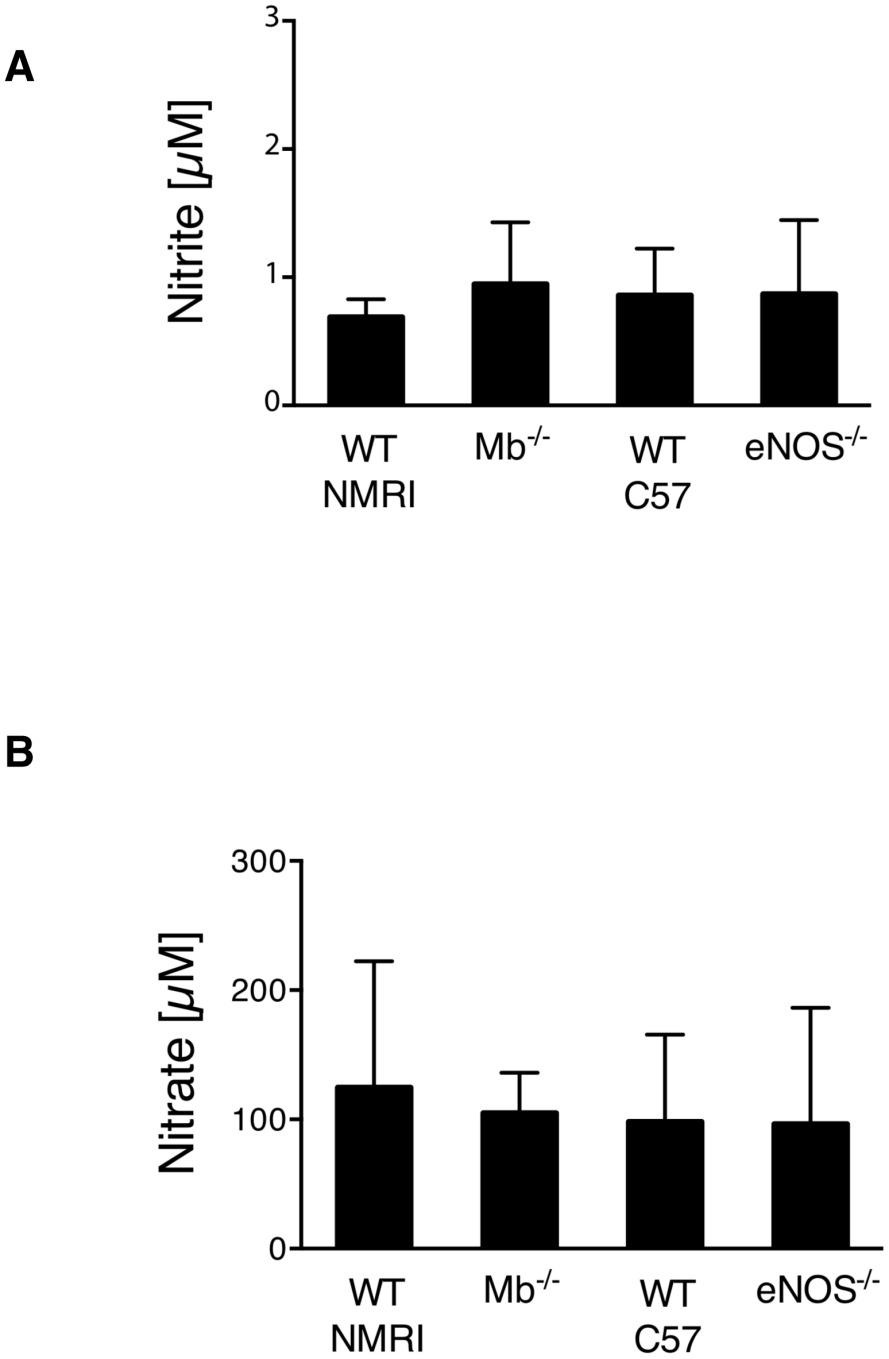
Nitrite and nitrate levels in mouse tissue. Aortic tissue of NMRI wild-types, myoglobin (Mb) deficient mice, C57BL/6 wild-types and endothelial nitric oxide synthase (eNOS) knockout mice was analyzed for (A) nitrite and (B) nitrate levels with no significant difference between the species as a prerequisite for dose-response experiments (n = 5–6, means±s.d.).

Tissue nitrite levels are generally believed to be more stable as compared to the circulation compartments. As a prerequisite for dose-response experiments it is necessary to demonstrate similar levels across the different species investigated. We show in **Figure 2a** that aortic tissue nitrite levels are not significantly different between NMRI wild-type and Mb-deficient mice ranging at approximately 1 µM. Moreover, no detectable differences were determined for eNOS-deficient mice and the C57Bl/6 wild-type mouse. Analysis of tissue nitrate levels is shown in **Figure 2b**. There were no detectable differences in nitrate concentrations between either mouse strain pointing to a selective increase of nitrite.

### Dose-dependent vasodilation by nitrite under hypoxia is Mb-dependent

**Figure 3:**
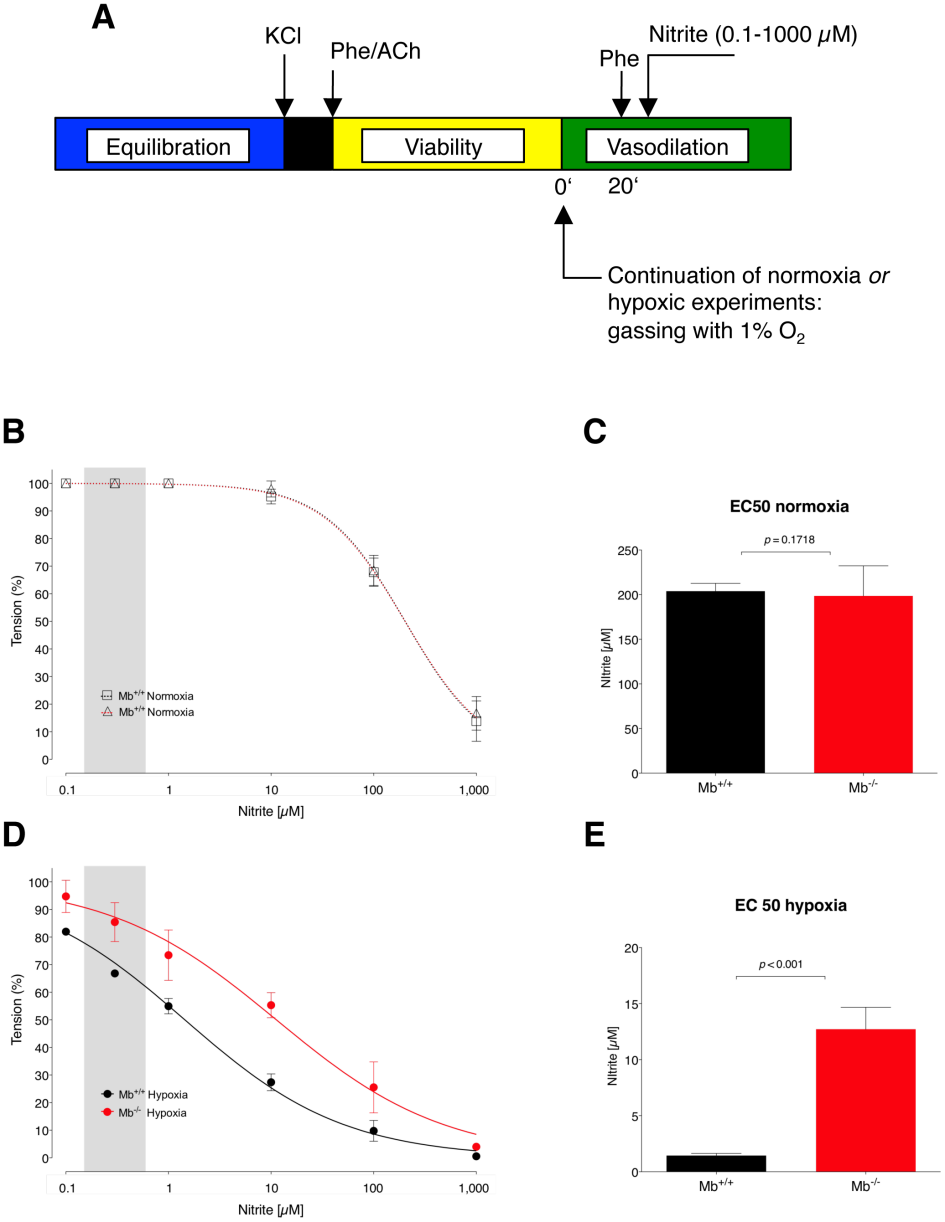
Dose-dependency for nitrite-induced hypoxic vasodilation in the presence and absence of myoglobin (Mb). (A) Experimental schema. After equilibration, normoxic gassing was either continued or changed to hypoxia (1% O_2_). Isolated aortic rings of *Mb^+/+^* and *Mb^−/−^* mice were then pre-constricted using phenylephrine (Phe) and subsequently challenged with cumulating doses of nitrite from physiological to pharmacological levels. Under normoxia, nitrite-vasodilation response were identical in both mouse types (B) leading to similar EC_50_ levels (C). On the contrary, under hypoxia, nitrite-induced vasodilation was significantly impaired in *Mb^−/−^* (D) with significantly higher resulting EC_50_ levels (E). All values are means±s.e.m.

The previous findings indicate that vascular tissue nitrite levels are similar in all investigated specimen under both normoxia and hypoxia. We then related these findings to vasodilation using an *ex vivo* bioassay to assess the potency of nitrite under hypoxia as a vasodilator of phenylephrine-constricted aortic rings for both *Mb^+/+^* and *Mb^−/−^* mice (experimental schema in **Figure 3a**). We found that aortic rings relaxed with increasing doses of nitrite from 0.1 to 1,000 µM under both normoxia and hypoxia (1% O_2_ or 7 mmHg). **Figure 3b and d** show the resulting dose/response curves. Under normoxia we found an identical dose/response in both *Mb^+/+^* and *Mb^−/−^* mice (**Figure 3b**), with a high half maximum concentration (EC_50_) of 204 µM (lower to upper limit: 176–213 µM) and 199 µM (lower to upper limit: 193–232 µM) nitrite, respectively, (*P* = 0.1718, **Figure 3c**). Hypoxia caused a substantial left shift of the dose/response curve, which was significantly potentiated in the *Mb^+/+^* compared with *Mb^−/−^* mice (**Figure 3d**), with calculated EC_50_s of 1.4 µM (lower to upper limit: 1.3–1.6 µM) and 12.7 µM (lower to upper limit: 8.7–14.7 µM, *P*<0.001, *n* = 5, **Figure 3e**), respectively. Thus, hypoxia induces a nitrite-dependent vasorelaxation that was substantially reduced in the *Mb^−/−^* mouse and effective from physiological to pharmacological nitrite concentrations.

### ROS modulate nitrite-induced vasodilation under hypoxia

**Figure 4:**
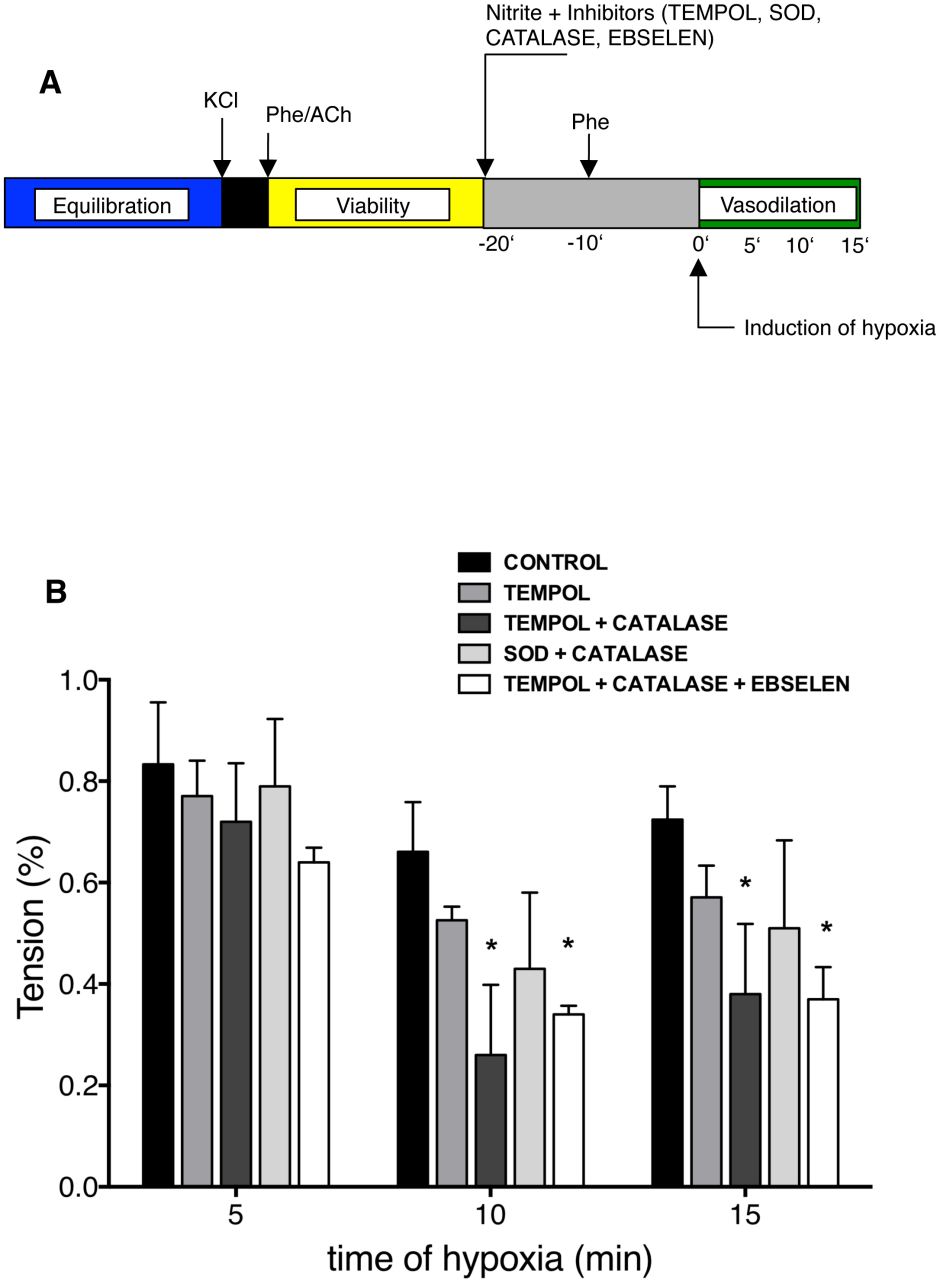
Endogenous ROS modulate the nitrite-induced hypoxic vasodilation response. (A) Experimental schema. After an equilibration period, SOD, SOD mimic tempol, catalase and gluthation peroxidase mimic ebselen were added to the organ bath in order to decompose endogenously formed ROS. The nitrite concentration in the organ bath was 300 nM. Vessels were then preconstricted with phenylephrine (Phe). After stabilization of constriction, hypoxic was induced and vasodilation observed for the following 15 min. (B) Graph shows the decrease in intention for the 15 min of hypoxia for controls and treated rings. Incubation of SOD mimic tempol with catalase and with catalase/ebselen significantly increased the vasodilation response at 10 and 15 min (*P<0.05, n = 3–5). Values are means±s.e.m.

It was recently demonstrated in penile artery preparations that ROS modulate relaxation under hypoxia. This pertained both to O_2_^•−^, whose breakdown is catalyzed by SOD, as well as to H_2_O_2_, whose decomposition is achieved through catalase, and peroxynitrite [34]. We here show that the elimination of O_2_^•−^ and H_2_O_2_ in parallel increased the vasodilatory properties of nitrite. Isolated mouse aortic rings were prepared and tested for viability as outlined above. Upon completion, control rings received a concentration of 300 nM of nitrite, which is a near physiological mouse plasma concentration. At this time point, rings were also challenged with ROS reducing agents. 10 min later, phenylephrine was used for pre-constriction and hypoxia was induced another 10 min later using the above outlined hypoxic conditions. Vasodilation was measured for the next 15 min (experimental schema in **Figure 4a**). To reduce ROS, rings were co-incubated with SOD-mimetic tempol to reduce O_2_^•−^ alone or in conjunction with catalase, which additionally reduces H_2_O_2_. Reduction of O_2_^•−^ increased the extent of vasodilation tendentially, this becomes significant when H_2_O_2_ is reduced in parallel at both the 10 and the 15 min time point (**Figure 4b**). Remarkably, we did not observe a significant increase in vasorelaxation when using SOD instead of SOD-mimetic tempol. This may be related to the different tissue penetration of these agents. No additional vasodilatory effect was observed when ebselen was used in combination with tempol and catalase to additionally reduce peroxynitrite. Taken together, the reduction of both O_2_^•−^ and H_2_O_2_ in parallel has a modulating effect on nitrite-induced vasodilation under hypoxia.

## Discussion

The key findings of the present study are that nitrite and Mb interact dose-dependently to induce vasodilation under hypoxia and that ROS modulate nitrite-induced vasodilation.

There is a growing interest in the biological properties of the inorganic anion nitrite and we and others have shown that nitrite plays a role in physiology and disease under hypoxic and ischemic conditions [4], [13], [14], [29], [46]–[50]. The majority of the hypoxic effects of nitrite have been related to its reduction to bioactive NO^•^. Heme proteins like Hb, Mb and members of the respiratory chain are mainly responsible for this reaction. Cosby and et al. were the first to show that this is relevant under near physiological nitrite conditions as demonstrated by a regulation of vascular functions in the presence of deoxygenated Hb [13]. It was furthermore demonstrated that exogenous nitrite causes a dose-dependent vasodilation and an increase in forearm blood flow in human volunteers at low to high pharmacological dosis [2], [45]. On the basis of these previous findings and taking advantage of the *Mb^−/−^* mouse, we recently extended this to baseline endogenous nitrite levels under physiological conditions [4]. Vascular Mb reacts with and reduces basal nitrite to NO^•^ within arterial vessel walls leading to vasorelaxation *ex vivo* and reduced blood pressure *in vivo*. This is accompanied by higher levels of NO^•^ as measured by electron spin resonance, nitrosylated Mb (MbNO), nitroso species (RSNO) and cGMP levels in wild-type as compared to *Mb^−/−^* mice implicating a higher NO^•^ production from nitrite in the presence of Mb. The availability of stable nitrite levels in the vasculature across all species as seen in our concurrent study may provide the basis for adequate nitrite-induced hypoxic vasodilation together with a demonstration that a moderate hypoxia, e.g. as seen in the working muscle, leads to a significant desaturation of Mb, which then reduces nitrite at much higher rates as compared to Hb in the circulation.

We next demonstrated that, across all nitrite concentrations, nitrite-induced vasodilation is significantly reduced in the absence of Mb with distinct EC_50_ values. This relates to physiological conditions (shaded grey the dose-response curves in **Figure 3**) as well as to higher pharmacological concentrations. In the context of our previous investigation aimed at the regulation of blood pressures under hypoxia [4], this argues in favor of a physiological role for nitrite in hypoxic vasodilation. On the other hand, nitrite may be used as a vasodilator with a bioactivity limited to ischemic and vulnerable tissues, e.g. in heart disease [29], pulmonary hypertension [51] and sickle cell disease [52]. Naturally, the vasodilatory effects of nitrite are modulated by a number of dependent or independent variables. It was demonstrated that acidic conditions favor the reduction of nitrite to NO^•^ with subsequently enhanced vasodilation [53]. This is also influenced by the extent of hypoxia and the availability of nitrite-reducing proteins. At O_2_ levels that cause a 50% desaturation of Hb, this heme globin is the main generator of NO^•^ from nitrite based on a maximum nitrite-reductase activity at half-saturation [13], [44]. With further decreasing O_2_ levels, other heme globins become relevant, e.g. Mb which accounts for up to 50% of the observed effects in our present investigations. Remarkably, Mb reduces nitrite much faster than other heme proteins. Under anoxic conditions, even eNOS is capable of producing NO^•^ from vascular tissue nitrite [21].

Finally, also ROS appear to contribute to the O_2_ sensing mechanisms involved in hypoxic vasodilation by limiting the extent of the vascular response. Earlier studies demonstrated that reduction of ROS in different vascular beds dramatically increases the reaction to hypoxia [34]. On the contrary, many cardiovascular diseases that contribute to vascular dysfunction are jointly characterized by an increased production of ROS with the specific contributors remaining under intensive debate. Quite consequently, hypoxic vasodilation was impaired in animal disease models with high ROS concentrations, e.g. in hypertension, the metabolic syndrome or obesity. Nitrite therapy, in turn, has been generally associated with a reduction of cardiovascular risk factors and disease [54]–[56]. This has been partially related to a modulation of mitochondrial respiration by reducing the extent of potentially harmful ROS [29], [57]. In the scope of hypoxic vasodilation, NO^•^ from nitrite-reduction as well as ROS may finally contribute to a balanced vasodilation response. Further studies will have to elucidate the exact source for ROS and for the interaction between nitrite-derived NO^•^ and ROS.

## Study Limitations

Hypoxia may occur when challenged with high altitude (e.g., our *in vivo* model simulates altitudes of 4000 meters), during exercise and during embryonic development. Vasodilation is an adaptive response to hypoxia occurring in conduit [13], [45], [58]–[60] and resistance-size arteries [8], [60]–[63] Isolated preparations of larger arteries are most commonly used to assess the underlying molecular mechanism that governs vasodilation under hypoxia [2], [13], [45], [64], [65]. The present findings implicate a major role for nitrite-reduction *via* Mb as well as ROS as modulators. However, due to the methodologies, the results should be regarded an approximation of the relevant vascular beds. Further studies in resistance vessels will be needed to fully elucidate the role of heme globins of the vessel wall, their nitrite reductase activity as well as the potential interactions with ROS.

## Conclusions

**Figure 5:**
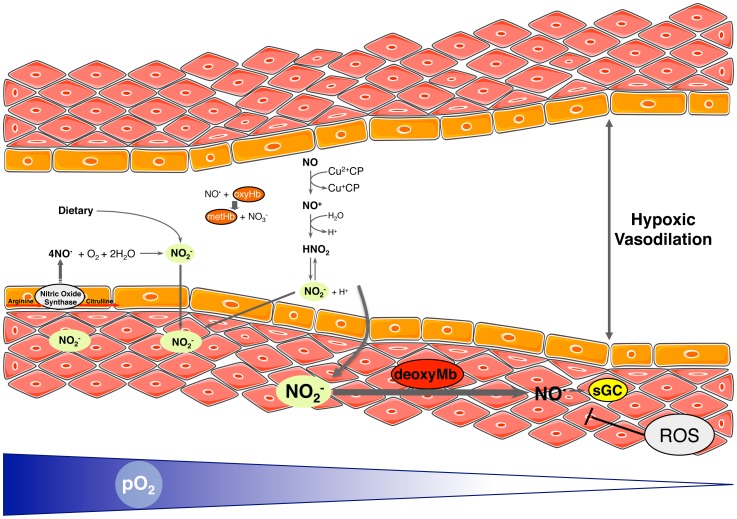
Role of nitrite and reactive oxygen species (ROS) in hypoxic vasodilation – proposed mechanism. Nitrite derives from NO^•^ synthesis dietary sources. NO^•^ to nitrite reactions occur by autoxidation or by reaction with ceruloplasmin (CP). Under hypoxia, nitrite levels in the vessel wall are increased. Nitrite can then be reduced to vasodilatory NO^•^ particularly by reaction with myoglobin (Mb). ROS modulate this response.

Under normoxia, eNOS generates NO^•^, which can act vasodilatory or as modulator of several other cardiovascular functions. Part of the generated NO^•^ then decomposes to nitrite, a reaction that is enhanced by circulating coeruloplasmin which is otherwise known for transporting copper. Decreasing O_2_ levels lead to Mb O_2_ unloading. This converts vascular Mb into a nitrite reductase, activating the vasodilatory machinery in a dose-dependent manner. ROS closely interact with NO^•^ and thus imfluence NO^•^ bioavailability. Here, we showed that the nitrite-induced vasodilation is much-enhanced under decreased ROS levels. Further studies, particularly measuring the exact levels of each ROS, are necessary to fully elucidate this interaction. **Figure 5** shows a schematic outline of the proposed mechanism.
